# Some Selected Cases of Amenorrhœa with Gross Lesion

**Published:** 1898-04-09

**Authors:** James Oliver

**Affiliations:** Physician to the Hospital for Women, London


### Medical Progress and Hospital Clinics.

[*The Editor will be glad to receive offers of co-operation and contributions from members of the profession. All letters should be addressed to* The Editor, at the Office, 28 & 29, Southampton Street, Strand, London, W.C.]

### SOME SELECTED CASES OF AMENORRHŒA WITH GROSS LESION.

By James Oliver, M.D., F.R.S.E., F.L.S., Physician to the Hospital for Women, London.

Case I. Parovarian cyst; operation; recovery.—Susan B., æt. 36, and married twelve years, has never been pregnant. During the last nine months there has been complete amenorrhœa, but prior to this the menstrual discharge had recurred regularly, and had been of the usual amount. In association with menstruation patient has always suffered much with backache, and for four or five days every month during the last nine months this symptom has returned, although the menstrual discharge has failed to make its appearance. For two years she has complained more or less of dull, aching pain in the left groin, and of late this has become more constant and more distressing. For twelve months there has been much white discharge from the vagina and frequent desire to pass water. Physical signs: There is nothing to note abdominally. The cervix uteri is located far back towards the hollow of the sacrum, and the os is slightly open. The vaginal roof to the left, and somewhat in front of the cervix, is pushed down by a globular and cystic swelling which is of about the size of a three months' pregnant uterus. The body of the uterus lies to the right of, and is definable from, this swelling. Operation: The abdominal cavity was opened mesially. After dealing with the omentum, which was extensively adherent to the anterior abdominal wall, the cyst, which was located in the left broad ligament, was tapped, and ten ounces of fluid were evacuated. The cyst was enucleated, and for twenty-four hours thereafter the cavity in the broad ligament was drained by a tube passing through the abdominal wound. The patient recovered without an untoward symptom.

Case II. Dermoid of right ovary; operation; recovery.—Ellen M., æt. 37, and married 10 years, has had one child, and this child was born six years ago, *i.e.*, four years after marriage. Since the birth of the child there has been complete amenorrhœa. For nine years patient has complained more or less of pain in the right iliac region. Physical signs: There is nothing to note abdominally. The cervix uteri is located fairly centrally. The right half of the pelvis is occupied by a small globular swelling of about the size of a cocoanut. To the right of the cervix the vaginal roof is somewhat puckered. Operation: The tumour, which was a dermoid in the right ovary, was removed by abdominal section. It had contracted adhesions with the omentum, and a portion of the latter structure was removed with the tumour. The uterus was much atrophied, and the left ovary could not be detected.

It is noteworthy that this patient had lived in wedlock three years before she conceived, and that she never menstruated after the birth of the child arising from this conception. The left ovary it would appear had never been evolved, and it is more than probable that the right had begun to degenerate before conception took place. After parturition, the periodic activity of the uterus was never aroused, and the organ atrophied in consequence of disuse.

Case III. Disease of both ovaries, fibroid transformation of the left, cystic transformation of the right; operation; recovery.—Ann B., æt. 30, and married six years, has never been pregnant. During the last two years menstruation has occurred very irregularly, sometimes after an interval of two or three months, and coetaneously the discharge has been greatly diminished. During these two years she has complained of a dull aching pain in the left iliac region which has generally been worst in the morning. Physical signs: There is nothing to note abdominally. The cervix uteri is rather low, and is located towards the right wall of the pelvis. The os looks forwards. The body of the uterus is felt posteriorly in Douglas's pouch verted. The vaginal roof on the left side, and somewhat anteriorly, is pushed down by a small, firm swelling, which is slightly irregular, and is of the size of a closed fist. This swelling is distinct from the uterus. The right ovary is larger than normal. Operation: The abdominal cavity was opened mesially. The tumour on the left side was found to be a solid fibroma of the left ovary, with the Fallopian tube coursing it. The mesovarium was so short that the tumour had to be enucleated to allow of efficient ligation. The right ovary, which was enlarged and in a state of cystic transformation, was also removed.

In this case irregular and scanty menstruation, and occasionally amenorrhœa of two or three months' duration, had been noted for two years. In association with fibroid transformation of one ovary, complete amenorrhœa is frequently observed. In the case of A. B., there was still some ovarian tissue in the right ovary, and it probably had only quite recently begun to undergo degenerative change, yet for two years amenorrhœa had occasionally been produced and the menstrual discharge had become scanty. In my opinion the menstrual disturbance was dependent solely upon the change which had taken place in the left ovary.

Case IV. Fibroma of right ovary; operation; regular recurrence of menstruation afterwards.—Janet A., æt. 27, and single, has not menstruated for fourteen months. About two years ago patient detected a small lump in the right iliac region close to the groin. It has gradually increased in size, but without causing pain. Physical signs: The patient is somewhat emaciated. The abdomen, which is very prominent, is occupied by a firm and regular swelling which extends from the pubes to the ensiform cartilage and well into both flanks. The cervix uteri is located centrally. The vaginal roof on the right side and slightly anteriorly is pushed down by a portion of the abdominal tumour. The body of the uterus lies behind, and is definable from the pelvic portion of the tumour. Operation: The abdominal cavity was opened mesially by a long incision, and, as there were no adhesions, the tumour was forthwith delivered. The mesovarium was short. The tumour weighed 7¾ lbs., and was as dense as cartilage. The patient made an excellent recovery. The menstrual discharge reappeared three weeks after the operation, and has recurred regularly ever since, *i.e.*, during a period of 12 months.

In this case the amenorrhœa was dependent upon the change which had taken place in the right ovary. The nutritive processes going on in the body had evidently been disturbed, and it is highly probable that the amenorrhœa was directly attributable to the metabolic disturbances, and that these in turn were due to the state of the right ovary. Three weeks after the removal of the morbid growth menstruation occurred, and it has continued (for 12 months) to recur regularly.

Case V. Adenoma (practically solid) of left ovary; operation; recovery.—Ellen I., æt. 23, and married two years, has never been pregnant. The menstrual discharge has always been scanty, but for 12 months there has been complete amenorrhœa. The abdomen during the last two years has gradually increased in size, and lately she has lost flesh. For two months she has complained of pain in the lower half of the abdomen, and in the back. For three or four months there has been frequent desire to pass water day and night. Physical signs: The body, generally, is badly nourished. The abdomen, which is very prominent, is occupied by a swelling which extends upwards from the pubes for 12 in. It is more prominent on the left side, and, although it extends to both flanks, it extends further into the left than the right. Crepitation is felt over its right upper half. With the exception of a spot on the right side, close to the umbilicus, measuring 3 in. by 3 in., where there is a sense of fluctuation, the tumour appears to be solid. The vaginal roof is peculiarly prolapsed, and drawn up into this prolapse is felt the cervix uteri; to the left of the cervix is felt a hard mass, which is a portion of the abdominal tumour; moving the abdominal tumour drags the cervix and the pelvic floor. Operation: The abdomen was opened mesially; the tumour was an almost solid adenoma, which had developed into the left broad ligament; it was enucleated, and the cavity in the broad ligament was drained for a few days by a tube passing through the abdominal wound; convalescence was tardy.

Case VI. Splenic Leuchæmia.—Annie H., æt. 25, and married three years, has never been pregnant, For six months there has been complete amenorrhœa. Prior to this menstruation had recurred regularly, and the discharge had been of the usual amount. During the last three months she has complained of morning sickness, and the abdomen has increased in size. Physical signs: The abdomen is prominent in the umbilical area. Above the umbilicus the abdomen is almost flat, and from three inches below the umbilicus it recedes markedly towards the pubes. The abdomen is occupied by a firm swelling, which extends well into the left flank, but the right border is felt 3½ in. to the right of the linea alba. Its lower border is felt 1½ in. above the pubes, and from this spot the tumour measures vertically 11 in. On the right margin of the tumour, at about the level of the umbilicus, is felt a notch. The uterus is retroverted. An examination of the blood revealed a great increase in the number of the white blood corpuscles.

On account of the cessation of menstruation, the morning sickness, and the increase in the size of the abdomen, this patient naturally was under the impression that she was pregnant, and it was to set her mind at rest on this point that she consulted me.

